# Mosses in Urban Environments as Passive Biofilters and Organisms Impacted by Asbestos-Contaminated Habitats

**DOI:** 10.3390/ijerph22060838

**Published:** 2025-05-26

**Authors:** Gergely Zoltán Macher, Dóra Beke

**Affiliations:** 1 Department of Applied Sustainability, Albert Kázmér Mosonmagyaróvár Faculty of Agricultural and Food Sciences, Széchenyi István University, 9026 Győr, Hungary; 2 Wittmann Antal Crop-, Animal- and Food Sciences Multidisciplinary Doctoral School, Albert Kázmér Mosonmagyaróvár Faculty of Agricultural and Food Sciences, Széchenyi István University, 9200 Mosonmagyaróvár, Hungary; 3 Department of Plant Sciences, Albert Kázmér Mosonmagyaróvár Faculty of Agricultural and Food Sciences, Széchenyi István University, 9200 Mosonmagyaróvár, Hungary; beke.dora@sze.hu

**Keywords:** asbestos cement, bryophytes, bioindicators, urban ecology, bioremediation

## Abstract

Asbestos cement materials represent a persistent source of environmental contamination, particularly in urban areas where weathering facilitates the release of hazardous chrysotile fibres. Despite extensive research on the human health impacts of asbestos, ecological interactions remain poorly understood. This paper explores the dual role of *bryophytes* colonising asbestos cement roofing as passive filters that trap airborne fibres and as vulnerable organisms subjected to asbestos-induced stress. Using a synthesis of recent findings, we assess the capacity of mosses to immobilise chrysotile fibres through their dense, mat-like structures, potentially reducing local dispersion. Simultaneously, we examine physiological and biochemical responses to prolonged fibre exposure, including reduced photosynthetic activity and signs of oxidative stress. The findings highlight a paradoxical function of *bryophytes*: while they contribute to pollution mitigation, they also accumulate contaminants and suffer from sublethal damage. These interactions may have broader implications for contaminant redistribution, particularly through decomposition and trophic transfer. Understanding these dynamics is essential for advancing ecological risk assessments and developing sustainable remediation strategies in asbestos-contaminated habitats.

## 1. Introduction

Asbestos cement (AC) roofing materials have been extensively employed worldwide since the early 20th century, particularly in the construction of industrial facilities, residential buildings, and public infrastructure [1,2,3]. Their widespread adoption can be attributed to their advantageous physical properties, including high tensile strength, low thermal conductivity, chemical stability, and fire resistance [4,5,6]. Moreover, AC products are cost-effective to produce and install, offering a practical solution for large-scale roofing and cladding applications [7]. Chrysotile, the most used form of asbestos, was especially favoured due to its fibrous flexibility and compatibility with Portland cement, allowing for the mass production of durable, lightweight roofing sheets [8]. Despite these functional advantages, the long-term health and environmental risks associated with asbestos-containing materials (ACMs) have rendered them a subject of growing concern and international regulatory action [9].

The inherent danger of asbestos lies in its fibrous, crystalline morphology. When disturbed—either through natural weathering, physical damage, or mechanical processing—AC materials can release microscopic fibres into the environment [10]. These fibres, particularly those in the respirable range (<10 µm in length), are capable of remaining airborne for extended periods, ultimately being inhaled or ingested by living organisms [11]. Once deposited in the lungs or other tissues, they can induce chronic inflammatory responses, genotoxic effects, and fibrotic changes, culminating in severe diseases such as asbestosis, lung carcinoma, and mesothelioma [12,13]. These adverse health effects are now well established and have prompted legislative bans, phase-outs, and large-scale remediation efforts in many countries.

However, while the human health implications of asbestos exposure are well documented, far less is known about the broader environmental consequences of asbestos degradation, particularly in relation to non-target organisms and ecosystem-level processes [14,15,16]. Asbestos fibres released into the environment can accumulate in soils, sediments, and surface waters, where they may persist for decades without significant degradation [17,18]. This knowledge gap is especially salient in urban and peri-urban ecosystems, where aging AC infrastructure continues to deteriorate in close proximity to both human populations and biologically active surfaces.

In recent years, growing attention has been directed toward the environmental pathways of asbestos contamination and the ecological interactions that may arise from chronic fibre exposure [19]. Research has begun to investigate the influence of asbestos on soil chemistry, microbial activity, and vegetative cover, yet these studies are still limited in scope and number [15]. In particular, the impact of asbestos fibres on non-vascular plants, such as *bryophytes*, has received little focused attention, despite their ecological importance and ubiquity in urban habitats [20]. These organisms frequently colonise inert surfaces, including AC roofing, where they may interact directly with deposited fibres [21]. Their structural simplicity and physiological sensitivity make them promising indicators of pollution, but also render them susceptible to environmental stressors [11].

In this context, it is imperative to broaden our understanding of how AC degradation contributes to diffuse environmental contamination [22,23], how this contamination manifests within ecological systems, and what roles organisms like mosses may play in modulating or exacerbating these effects [24,25]. This review aims to synthesise the current state of knowledge on asbestos-related environmental dynamics, with a particular focus on the colonisation of AC surfaces by *bryophytes*, their potential to retain chrysotile fibres, and the physiological impacts of prolonged exposure. By highlighting this emerging field of research, we seek to inform future investigations and contribute to the development of integrated monitoring and management strategies in asbestos-contaminated environments.

This review furnishes a novel contribution through the synthesis of disparate strands of the ecological, toxicological, and environmental health literature, thereby exploring the dual role of mosses on asbestos cement surfaces. Unlike prior studies that focus narrowly on asbestos toxicity exclusively, this manuscript integrates the horizontal thematic areas domains to critically assess mosses both as physiological responders and as functional agents in fibre immobilisation. Furthermore, the paper identifies overlooked knowledge gaps and proposes future research directions, thus serving as a conceptual and methodological platform for interdisciplinary investigation.

## 2. Urban Ecological Fate of Asbestos

The environmental persistence and complex behaviour of asbestos fibres following their release from aging and deteriorating AC materials represent a significant, yet insufficiently characterised, challenge in the context of urban ecological health [14,15]. Once these fibres are liberated—primarily through mechanical abrasion, freeze-thaw cycles, photodegradation, or biogenic activity—they enter environmental matrices where they can be transported across spatial and ecological boundaries [26,27]. Aeolian dispersion enables asbestos fibres to become airborne and settle over considerable distances from their original point of release, while surface runoff and leaching during precipitation events facilitate their entry into soil horizons, drainage systems, and surface waters [28,29,30]. In addition to abiotic vectors, biotic agents such as animals or vegetative detritus may further contribute to the redistribution and accumulation of fibres in previously unimpacted zones [31].

Upon deposition, asbestos fibres exhibit exceptionally low biodegradability and chemical weathering potential due to their silicate-based mineral composition, which includes serpentine or amphibole forms [32,33]. This chemical inertness renders asbestos an archetype of a persistent inorganic pollutant, capable of maintaining its structural integrity and toxicological potential over extended timescales [13,19,34]. In terrestrial environments, fibres can embed within soil aggregates, forming physical barriers that disrupt water movement and alter microhabitat structures [14]. In aquatic ecosystems, they may adsorb to sediment particles or remain in suspension, depending on local pH, ionic strength, and hydrodynamic conditions [35,36,37]. These mechanisms influence the bioavailability of asbestos and its potential for trophic transfer, yet current models for predicting fibre fate and transport remain rudimentary [38].

Beyond mere physical persistence, emerging research has begun to document the subtle but consequential impacts of asbestos contamination on biological communities [39,40]. Experimental studies suggest that asbestos fibres can interfere with microbial activity by disrupting cell membranes, altering enzyme kinetics, or modifying community composition [41,42,43]. Such disruptions can have cascading effects on nutrient cycling, soil respiration, and organic matter decomposition, all of which are critical for ecosystem functioning [44,45,46]. Invertebrate fauna, especially detritivores and burrowing organisms, may also be exposed to fibres via ingestion or dermal contact, leading to behavioural and physiological impairments that reverberate through food web structures [47].

These ecological effects are especially concerning in urban and peri-urban areas, where remnant AC infrastructure remains prevalent and intersects directly with biologically active surfaces [8,48]. Such environments are characterised by high degrees of anthropogenic disturbance, fragmented habitats, and frequent human–wildlife interactions, creating conditions under which pollutant exposure may be intensified [49]. However, systematic ecological assessments of asbestos in these contexts are exceedingly rare. There is a notable paucity of field-based studies quantifying fibre loads in soil and vegetation, assessing organismal exposure routes, or evaluating sublethal effects across taxonomic groups [15].

One particularly understudied yet ecologically relevant domain concerns the interactions between asbestos fibres and cryptogamic organisms, such as *bryophytes* and lichens, which are among the earliest colonisers of exposed AC surfaces [16]. These non-vascular plants often form extensive mats that directly intercept airborne particles, including asbestos. Due to their poikilohydric physiology and absence of protective cuticles, *bryophytes* are particularly susceptible to external environmental stressors, making them both potential sinks and sentinels for asbestos pollution [50,51,52]. Nevertheless, research into the mechanistic underpinnings of bryophyte–asbestos interactions remains scant [53]. Studies investigating whether asbestos fibres alter *bryophyte* physiology, affect photosynthetic efficiency, or contribute to intracellular oxidative stress are urgently needed to assess the long-term ecological consequences of fibre colonisation on these foundational organisms [54,55].

## 3. Bryophytes as Early Colonisers and Indicators of Pollution

*Bryophytes*—comprising mosses, liverworts, and hornworts—are among the earliest-diverging lineages of terrestrial plants and exhibit a unique suite of physiological, morphological, and ecological traits that enable them to thrive in a wide array of environments, including urban and industrial habitats [56,57]. These non-vascular plants are particularly notable for their capacity to colonise nutrient-poor and structurally challenging substrates, such as exposed rock surfaces, concrete walls, asphalt pavements, and degraded AC roofing [54,58,59]. Their status as pioneering colonisers stems from their low resource requirements, high reproductive efficiency through vegetative propagation, and exceptional desiccation tolerance [60]. In urbanised landscapes where extreme temperature fluctuations, limited moisture retention, and chemical pollution prevail, *bryophytes* frequently constitute the first biotic layer capable of establishing persistent populations [53,61].

A key feature of *bryophyte* ecology is their poikilohydric nature; that is, their water content equilibrates rapidly with that of the surrounding environment [24]. This characteristic, along with their high surface area-to-volume ratio and absence of protective cuticular layers or complex vascular systems [62], facilitates the direct and often unregulated uptake of water, nutrients, and pollutants from both the atmosphere and the substratum [50,63,64]. Over the past decades, they have been extensively utilised to assess atmospheric deposition of nitrogen compounds, sulphur oxides, persistent organic pollutants (POPs), and—most prominently—heavy metals such as lead, cadmium, zinc, and mercury [65,66,67,68,69,70,71].

*Bryophytes* accumulate contaminants via both wet and dry deposition, facilitated by their simple anatomy and large extracellular cation exchange capacity [53,72]. Unlike vascular plants, *bryophytes* do not possess roots or an internal circulatory system, meaning that most of their nutrient and pollutant acquisition occurs via surface absorption [73]. This makes them highly effective recorders of ambient pollution levels, and in turn, valuable tools for long-term environmental monitoring. Bryophyte-based biomonitoring offers several advantages, including low cost, ease of sampling, wide geographic applicability, and the ability to track spatial and temporal pollution gradients with high resolution [66,74,75]. As summarised in Table 1, mosses possess a combination of physiological and ecological traits that may render them particularly suitable for asbestos fibre interception compared to other bioindicators.

Field observations and limited surveys suggest that certain moss genera, such as *Hypnum*, *Bryum*, and *Dicranum,* are frequently encountered on asbestos cement surfaces, particularly in temperate urban environments [60,76]. These species’ structural characteristics and tolerance to anthropogenic substrates may make them particularly relevant for future asbestos biomonitoring applications [77]. However, detailed floristic studies are required to determine community composition patterns in relation to asbestos contamination levels.

While the focus of this review centres upon *bryophytes*, it is indeed worth noting that other biological groups have likewise been employed as bioindicators in studies concerning pollution. Such groups include lichens, algae, and vascular plants.

However, in the specific context of asbestos cement surfaces, only *bryophytes* and lichens appear to establish consistently and in ecologically meaningful densities. Algae and vascular plants, though theoretically valuable in other settings, are seldom observed colonising such substrates due to their requisite needs for sustained moisture, nutrients, or soil. The same properties that make *bryophytes* excellent indicators of pollution also predispose them to physiological damage when exposed to elevated levels of contaminants [78]. Accumulated toxicants can interfere with key physiological processes, such as photosynthesis, respiration, and protein synthesis. For instance, heavy metals and fine particulate matter may disrupt chloroplast function, alter cellular osmotic balance, and induce the production of reactive oxygen species (ROS), leading to oxidative stress [79,80,81]. Moreover, the interaction between pollutants and *bryophyte* tissues is often mediated by complex biochemical pathways that may vary among species, developmental stages, and environmental contexts, complicating the interpretation of biomonitoring data [72,82,83,84].

In addition to their physiological sensitivity, *bryophytes* also play a functional ecological role in urban ecosystems by modulating microclimatic conditions, promoting substrate stability, and influencing biogeochemical cycling at localised scales [72,82,85]. On AC roofs, their dense, mat-like growth may reduce surface temperatures and buffer moisture availability, potentially slowing the physical weathering of the substrate. Simultaneously, this growth form enhances their capacity to trap airborne particulates, including asbestos fibres, heavy metals, and microplastics [29,86]. The extent to which these passive filtration processes contribute to contamination retention versus biological harm, however, remains underexplored. *Bryophytes* colonising asbestos-containing surfaces may be subject to dual pressures: acting as pollutant sinks while experiencing chronic exposure to structurally invasive and chemically inert fibrous particles [24,87,88]. Despite their widespread use as biomonitors, *bryophytes* are often excluded from ecological risk assessments and pollution mitigation frameworks. This oversight is especially significant in urban and industrial landscapes, where *bryophyte* communities frequently represent the dominant autotrophic component in microhabitats devoid of vascular vegetation [89,90,91]. A more nuanced understanding of how *bryophytes* respond to and interact with environmental pollutants—particularly inorganic particulates like asbestos—is essential for integrating cryptogamic flora into sustainable urban ecological planning, remediation strategies, and environmental health surveillance systems [53,82,92].

## 4. Moss–Asbestos Interactions and Physiological Effects

**Figure 1:**
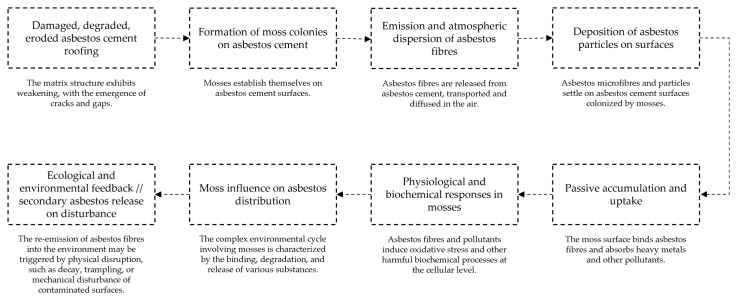
Pathway of moss–asbestos interactions.

Mosses growing on AC substrates are not merely passive settlers of anthropogenic surfaces; rather, they engage in multifaceted interactions with the material, especially with the chrysotile fibres that become exposed through weathering [14,93,94]. These interactions involve both mechanical entrapment and physiological contact (Figure 1), positioning mosses simultaneously as passive filters of environmental pollutants and as biological entities vulnerable to fibre-induced stress [95,96]. The ability of mosses to retain asbestos fibres primarily derives from their dense [29,97], mat-forming growth habit and high surface area, which promote the interception and immobilisation of airborne particles [11,29,95]. The surface morphology of mosses—particularly the leaf and stem surfaces coated in mucilage and rich in extracellular binding sites—facilitates the adhesion of mineral particles. Once intercepted, fibres may become embedded in the apoplast or entrapped within the mat, where they are subject to minimal physical displacement. This passive fibre retention potentially mitigates the dispersal of airborne asbestos particles, contributing to localised containment and reduced exposure risk for humans and other organisms in the vicinity [16].

However, this retention is not without biological cost. Experimental and observational studies indicate that asbestos fibre accumulation may elicit significant physiological stress in *bryophytes*, though the precise mechanisms remain underexplored [13,16]. Evidence suggests that exposure to chrysotile fibres is associated with reductions in chlorophyll content, impaired photosynthetic efficiency, and alterations in cell membrane permeability, all of which point to disruption of cellular homeostasis [13,38]. These effects are likely exacerbated by the fibres’ needle-like morphology and high surface reactivity, which may induce mechanical damage at the tissue level and facilitate the generation of reactive oxygen species. Prolonged or repeated exposure may overwhelm mosses’ antioxidant defences, leading to oxidative stress, lipid peroxidation, and eventual cell death [98,99,100]. Furthermore, the chemical leaching of elements from degraded AC—such as magnesium, calcium, or even trace heavy metals—may contribute additional stress factors. These compounds can alter local pH conditions and ionic balances, further affecting moss physiology. The combined influence of physical abrasion, chemical toxicity, and pollutant accumulation positions AC surfaces as ecologically challenging environments, even for *bryophytes* adapted to extreme conditions [54,101,102].

Complicating this picture is the possibility of secondary interactions between the retained asbestos fibres and other environmental contaminants. Mosses are known to accumulate a broad spectrum of pollutants, and the presence of chrysotile may modify the uptake, sequestration, or toxicity of co-occurring substances such as heavy metals or hydrocarbons [23,103]. These synergistic or antagonistic effects are poorly understood but may have profound implications for moss viability and for the interpretation of biomonitoring data collected from AC surfaces [104]. Investigating these responses at the molecular and population levels could yield insights into adaptive strategies under chronic contamination, such as the upregulation of stress-response genes, modifications in cell wall composition, or symbiotic interactions with protective microbial communities [105,106]. Although the general physiological impacts of asbestos exposure—such as photosynthetic inhibition and oxidative stress—are noted, more detailed investigation is warranted [107]. Potential biochemical biomarkers include antioxidant enzyme activities (superoxide dismutase (SOD), catalase (CAT), and peroxidase (POD)), and lipid peroxidation markers such as malondialdehyde (MDA) [108,109]. These indicators may provide quantitative insight into stress responses at the cellular level. Additionally, omics-based techniques, including transcriptomics and metabolomics, could illuminate molecular pathways underlying *bryophyte* tolerance or adaptation to asbestos-contaminated substrates [95,110,111].

Understanding the extent to which mosses can mitigate environmental fibre dispersal while maintaining ecological function is essential for integrating them into nature-based solutions for passive remediation [25,112,113]. Moreover, elucidating the physiological pathways through which mosses respond to asbestos exposure may reveal bioindicators of fibre presence, with potential applications in non-invasive contamination mapping and urban ecosystem health monitoring [114,115]. From a practical perspective, the employment of mosses as a passive containment layer on asbestos-containing surfaces presents a low-technology, potentially scalable approach to fibre stabilisation. Nonetheless, its success hinges upon multiple factors, including moss species selection, substrate compatibility, climatic resilience, and maintenance requirements. Case studies or pilot projects that assess long-term moss viability and fibre retention under real-world stressors are critically essential to validate this concept as a viable remediation tool.

## 5. Implications for Urban Ecology and Asbestos Risk Management

The colonisation of AC surfaces by mosses and their subsequent interactions with asbestos fibres have far-reaching consequences for urban ecological dynamics and environmental risk management [29]. In densely built environments, where AC roofing remains prevalent—particularly in aging infrastructures and low-income areas—the presence of *bryophyte* mats may exert a dual effect; they may act as biological barriers to fibre dispersion, while simultaneously becoming vectors of fibre accumulation and redistribution through biotic and abiotic processes [95,116]. From an ecological perspective, moss colonisation introduces a biotic interface to otherwise inert, mineralised urban surfaces. This green layer fosters microhabitats, influences local microclimates, and serves as an anchor for successional processes, often facilitating the establishment of other organisms, including algae, fungi, and invertebrates [117,118,119]. However, when the substrate is asbestos-laden, this ecological scaffolding becomes entangled with pollution dynamics [43]. Mosses that retain chrysotile fibres on their surfaces or within their tissues may inadvertently introduce fibres into new trophic levels through herbivory, detrital cycling, or bioturbation. Although direct evidence remains scarce, such pathways could expand the ecotoxicological footprint of asbestos far beyond its point of origin, affecting organisms not directly exposed to the material itself [16,120,121].

Moreover, the persistence of mosses on AC roofs underscores a critical paradox: their ecological functions may mask the presence of hazardous materials from public awareness. The visual greening of urban rooftops may suggest environmental revitalisation, while it could signal the development of biologically active but contaminated microecosystems [122,123,124]. This camouflage effect complicates risk communication and calls for more nuanced approaches in urban environmental monitoring, where visual cues must be complemented by analytical surveillance, including microscopic and chemical analyses of retained fibres [125,126]. From a risk management perspective, the ability of mosses to immobilise asbestos fibres introduces opportunities for developing nature-based remediation strategies [127]. Passive fibre retention by moss mats may be harnessed as a low-cost, low-impact interim solution for mitigating airborne asbestos release, particularly in contexts where full removal or encapsulation of AC materials is economically or logistically unfeasible. Such an approach, however, would require a detailed understanding of moss–fibre interaction dynamics, long-term physiological effects on the moss community, and the potential for re-aerosolisation of trapped fibres under dry or disturbed conditions [25,95].

The dual role of mosses also highlights the need to integrate ecological monitoring with public health frameworks. Moss communities may serve as early warning systems for asbestos degradation, with shifts in species composition, pigment content, or cellular structure offering non-invasive indicators of contamination [128,129]. However, the deployment of *bryophytes* as biomonitors must account for their physiological limits and potential feedback effects, such as fibre accumulation altering their pollutant-uptake profiles or competitive interactions with other organisms [48,54,72,75]. Urban planners, public health authorities, and environmental scientists must therefore recognise mosses not merely as incidental colonisers of AC surfaces, but as active agents in the urban asbestos cycle. Incorporating their presence into risk assessment models, remediation protocols, and urban green infrastructure planning may offer new avenues for holistic environmental management [25,60,130]. Yet this requires a paradigm shift, from viewing mosses as passive recipients of pollution to acknowledging their complex role in modulating contaminant pathways, structuring microecological processes, and mediating human-environment interactions in polluted urban spaces [25,131].

## 6. Conclusions

The intricate interplay between mosses and AC surfaces highlights the dual ecological and toxicological roles these *bryophytes* occupy in contaminated urban environments. Far from being incidental colonisers of deteriorating infrastructure, mosses function as both biological filters capable of immobilising hazardous fibres and as living organisms susceptible to fibre-induced physiological stress. This duality challenges traditional perceptions of mosses as benign or passive elements in the urban landscape and positions them as active agents in the modulation of asbestos dispersion and toxicity.

The ability of mosses to trap chrysotile fibres through their dense mat structures and mucilage-coated surfaces presents a potentially valuable function in the context of passive pollution mitigation. Particularly in low-income or aging urban areas where asbestos removal is economically or logistically impractical, moss colonisation may offer a form of low-impact, interim fibre containment.

However, this capacity comes at a biological cost: the accumulation of asbestos fibres within moss tissues has been associated with reductions in photosynthetic efficiency, oxidative stress responses, and eventual cell damage or death. These findings raise important questions about the long-term viability of moss populations under chronic exposure conditions and the sustainability of relying on such systems for remediation without further intervention.

Although promising as a nature-based solution, the real-world implementation of moss-based asbestos mitigation strategies necessitates critical evaluation. Are moss treatments on asbestos roofs feasible on a larger scale? Can moss mats persist under environmental stressors such as drought, ultraviolet radiation, and mechanical disturbance? How would seasonal changes or urban maintenance practices impact their stability and retention function? Pilot studies and in situ experiments are urgently required to ascertain whether these moss-covered surfaces can serve as sustainable, low-maintenance containment systems or if they necessitate periodic renewal, supplementation, or protective management. In the absence of this evidentiary foundation, practical deployment remains speculative.

Moreover, the presence of mosses on AC surfaces may inadvertently contribute to the redistribution of asbestos fibres through ecological processes such as herbivory, detrital decomposition, or bioturbation. This potential for trophic and spatial transfer expands the conceptual footprint of asbestos contamination, indicating that the risk is not confined solely to direct human inhalation or dermal contact but may propagate through broader ecosystem pathways.

Compounding this complexity is the possibility of synergistic toxicity with co-occurring pollutants such as heavy metals or hydrocarbons, the effects of which are still poorly understood in the context of *bryophyte* physiology and pollutant bioavailability.

From a risk communication and management perspective, the persistence of moss mats on asbestos-containing surfaces may create a form of ecological camouflage. Visually, such surfaces may appear to be undergoing natural greening or ecological recovery, masking the underlying presence of a hazardous material. This disjunction between appearance and reality necessitates more nuanced public health messaging and integrated environmental monitoring approaches that go beyond surface-level assessments and incorporate microscopic, chemical, and physiological indicators of contamination.

Looking forward, the integration of mosses into urban asbestos risk management frameworks demands a paradigm shift, from viewing these organisms as passive recipients of pollution to recognising them as dynamic bioindicators and ecosystem engineers.

Future research should prioritise molecular and ecophysiological investigations into moss tolerance mechanisms, including stress-related gene expression, cell wall adaptations, and potential mutualistic interactions with microbiota. Such insights may inform the development of targeted biomonitoring protocols and enhance the efficacy of nature-based remediation strategies. Bridging ecological function with toxicological risk, mosses occupy a unique niche at the intersection of natural resilience and anthropogenic hazard—one that merits closer examination in the pursuit of sustainable urban environmental health.

As the existing literature on moss–asbestos interactions remains sparse and largely descriptive, this review aims to establish a foundation for systematic research. The identification of physiological stress markers, the mapping of species-specific colonisation patterns, and the feasibility of large-scale moss deployment for asbestos containment all represent critical avenues for further investigation. By highlighting these gaps, the manuscript invites targeted experimental work and cross-disciplinary collaboration aimed at validating and extending the ecological applications of *bryophytes* in contaminated urban environments.

## Figures and Tables

**Table 1:** Comparative overview of common bioindicator groups used in pollution monitoring.

Organism Group	Key Advantages	Key Limitations	Relevance to Asbestos Cement Surfaces
Mosses	High surface-area-to-volume ratio, poikilohydric, survive in extreme substrates	Sensitive to desiccation in exposed sites	Frequently colonise asbestos cement, trap fibres
Lichens	Long lifespan, good for long-term accumulation studies	Slow growth, sensitive to sulphur and pH extremes	Less common on urban asbestos cement surfaces
Algae	Rapid growth, useful for waterborne pollutants	Require moisture/nutrients, less substrate specificity	Rare on dry or vertical asbestos cement surfaces
Vascular plants	Structural root systems, visible physiological changes	Limited to well-developed soils, slower colonisation	Rare on intact AC surfaces, more relevant post-weathering
